# Enhancing liver cirrhosis varices and CSPH risk prediction with spleen stiffness measurement using 100-Hz probe

**DOI:** 10.1038/s41598-024-63848-5

**Published:** 2024-06-13

**Authors:** Jeong-Ju Yoo, Sun Ah Maeng, Young Chang, Sae Hwan Lee, Soung Won Jeong, Jae Young Jang, Gab Jin Cheon, Young Seok Kim, Hong Soo Kim, Sang Gyune Kim

**Affiliations:** 1 Division of Gastroenterology and Hepatology, Department of Internal Medicine, Soonchunhyang University Bucheon Hospital, 170 Jomaru-ro, Bucheon, 14854 Republic of Korea; 2 Department of Internal Medicine, Soonchunhyang University Seoul Hospital, Seoul, Republic of Korea; 3 Department of Internal Medicine, Soonchunhyang University Cheonan Hospital, Cheonan, Republic of Korea; 4 Department of Internal Medicine, Gangneung Asan Hospital, Gangneung, Republic of Korea

**Keywords:** Spleen stiffness, Varices, Liver cirrhosis, Fibroscan 630, Portal hypertension, HVPG, Hepatology, Liver

## Abstract

Managing complications of liver cirrhosis such as varices needing treatment (VNT) and clinically significant portal hypertension (CSPH) demands precise and non-invasive diagnostic methods. This study assesses the efficacy of spleen stiffness measurement (SSM) using a 100-Hz probe for predicting VNT and CSPH, aiming to refine diagnostic thresholds. A retrospective analysis was conducted on 257 cirrhotic patients, comparing the diagnostic performance of SSM against traditional criteria, including Baveno VII, for predicting VNT and CSPH. The DeLong test was used for statistical comparisons among predictive models. The success rate of SSM@100 Hz was 94.60%, and factors related to SSM failure were high body mass index and small spleen volume or length. In our cohort, the identified SSM cut-off of 38.9 kPa, which achieved a sensitivity of 92% and a negative predictive value (NPV) of 98% for detecting VNT, is clinically nearly identical to the established Baveno threshold of 40 kPa. The predictive capability of the SSM-based model for VNT was superior to the LSM ± PLT model (*p* = 0.017). For CSPH prediction, the SSM model notably outperformed existing non-invasive tests (NITs), with an AUC improvement and significant correlations with HVPG measurements (obtained from 49 patients), highlighting a correlation coefficient of 0.486 (*p* < 0.001) between SSM and HVPG. Therefore, incorporating SSM into clinical practice significantly enhances the prediction accuracy for both VNT and CSPH in cirrhosis patients, mainly due to the high correlation between SSM and HVPG. SSM@100 Hz can offer valuable clinical assistance in avoiding unnecessary endoscopy in these patients.

## Introduction

Significant advancements have been made in the management of varices, with preventive measures and early interventions being key pillars in reducing variceal bleeding-related morbidity and mortality^1^. Traditionally, upper gastrointestinal endoscopy has been the gold standard for diagnosing varices and assessing their severity. However, this invasive procedure has drawbacks, including patient discomfort, associated risks, and limited accessibility in certain healthcare settings^2^. In recent years, non-invasive techniques for predicting varices needing treatment (VNT) have emerged as promising alternatives. Among these novel approaches, spleen stiffness assessment has garnered increasing attention. The spleen plays a pivotal role in the pathophysiology of portal hypertension, acting as a blood reservoir, and alterations in its stiffness have been linked to the presence and severity of varices^3^.

Previous reports have demonstrated that measuring spleen stiffness provides valuable insights into the dynamics of portal hypertension^4,5^. By integrating spleen stiffness evaluation with LSM and other clinical parameters, clinicians can gain a more comprehensive understanding of the patient's liver disease and their risk of developing varices. These non-invasive techniques have contributed to the recent publication of the Baveno VII criteria, which suggest a cut-off of spleen stiffness at 40 kPa for screening VNT^6^. However, spleen stiffness measurement (SSM) using 2D-shear wave elastography (SWE) and point-SWE has shown a higher failure rate compared to liver stiffness measurement (LSM) and has the disadvantage of varying cut-offs depending on the machine. Additionally, due to the spleen's inherent stiffness relative to the liver, there is a limitation in potentially overestimating spleen stiffness when performing SSM with an SSM@50 Hz probe used for LSM measurement. Therefore, a novel spleen-dedicated probe using SSM@100 Hz has recently been developed, demonstrating higher accuracy than the existing SSM@50 Hz^7^.

The purpose of this study is to investigate whether SSM using the SSM@100 Hz probe can predict VNT and clinically significant portal hypertension (CSPH), and to explore its correlation with hepatic venous pressure gradient (HVPG) in patients with liver cirrhosis. Additionally, the study aims to identify factors associated with the failure of SSM.

## Results

### Patients’ characteristics

**Figure 1:**
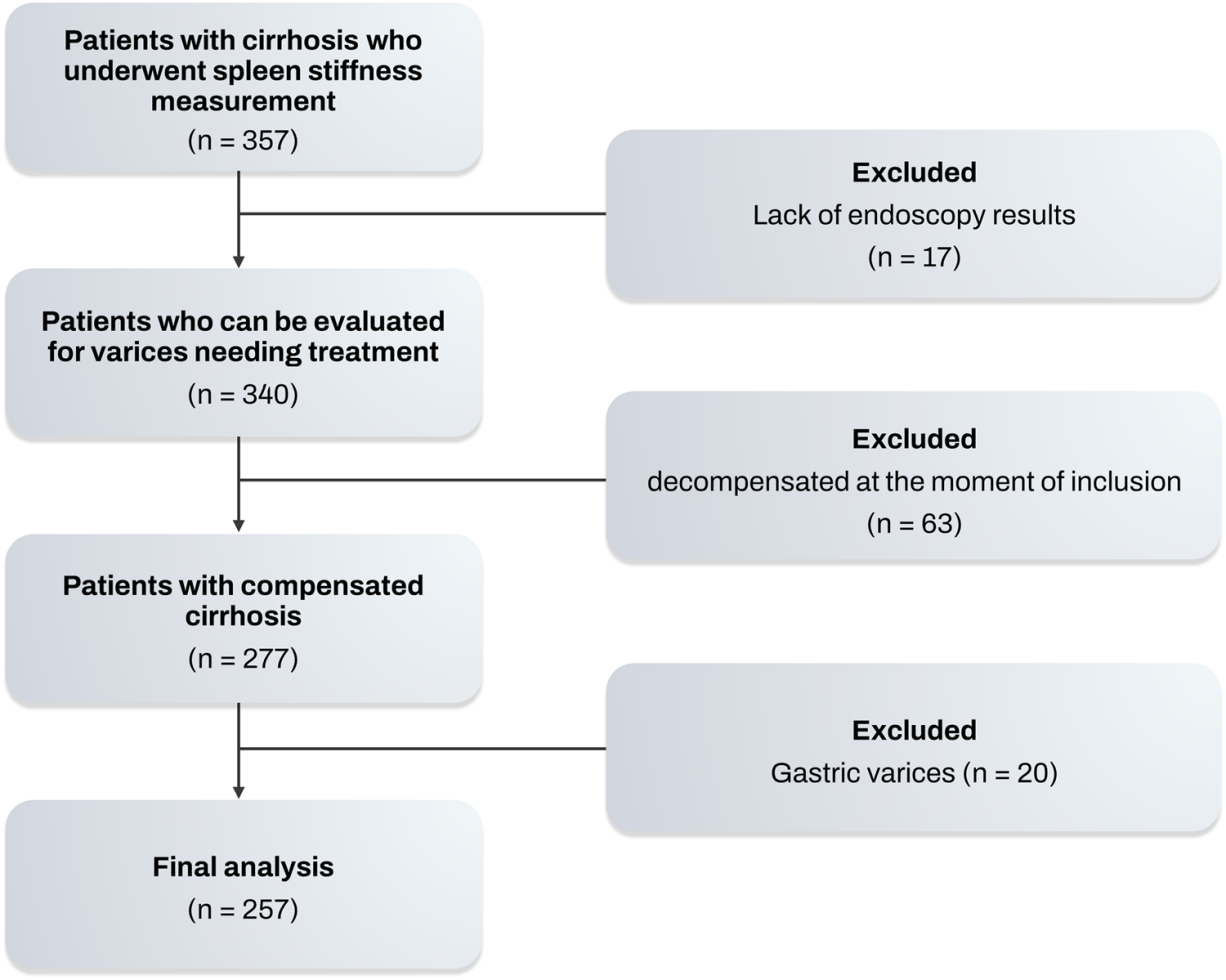
Flow chart.

Finally, 257 patients were selected for analysis (Fig. 1). The baseline characteristics of the 257 patients analyzed in the study are presented in Table 1. The most common etiology of liver disease was HBV (40.08%), followed by alcohol (34.63%). All patients with a viral etiology (HBV or HCV) had achieved viral suppression through antiviral treatment. VNT was detected via endoscopy in 52 (20.23%) of the patients. The group with VNT (+) exhibited significantly greater spleen length, spleen volume, LSM, and SSM compared to the VNT (−) group (Table 1).

**Table 1:** Baseline characteristics of patients.

	Total (N = 257)	VNT (−) (N = 205)	VNT (+) (N = 52)	*P*
Age (years)	59.14 ± 10.64	59.42 ± 10.92	58.02 ± 9.47	0.396
Sex, male	152(59.14%)	124(60.49%)	28(53.85%)	0.384
Etiology				0.223
HBV	103(40.08%)	86(41.95%)	17(32.69%)	
HCV	16(6.23%)	15(7.32%)	1(1.92%)	
Alcohol	89(34.63%)	65(31.71%)	24(46.15%)	
NAFLD	8(3.11%)	7(3.41%)	1(1.92%)	
Others	41(15.95%)	32(15.61%)	9(17.31%)	
Body mass index (kg/m^2^)	24.62 ± 3.44	24.62 ± 3.45	24.62 ± 3.40	0.989
Spleen stiffness measurement fail	15(5.84%)	14(6.83%)	1(1.92%)	0.178
Spleen length (mm)	109.28 ± 23.72	104.11 ± 20.30	130.03 ± 25.30	< 0.001
Spleen volume (cm^3^)	332.54 ± 234.94	279.74 ± 182.89	544.78 ± 296.06	< 0.001
Spleen stiffness (kPa)	35.08 ± 18.82	29.46 ± 15.12	56.12 ± 16.29	< 0.001
Liver stiffness (kPa)	18.83 ± 16.39	15.83 ± 14.11	30.81 ± 19.34	< 0.001
HVPG (mm Hg) (N = 49)	13.41 ± 4.73	12.76 ± 5.28	14.35 ± 3.73	0.251
Platelet count (× 10^3^/mL)	146.75 ± 74.49	158.84 ± 74.69	99.77 ± 52.12	< 0.001
AST (U/L)	42.08 ± 38.27	41.69 ± 40.72	43.62 ± 26.74	0.746
ALT (U/L)	27.29 ± 31.35	28.52 ± 34.39	22.44 ± 13.11	0.212
Total bilirubin (mg/dL)	1.26 ± 0.85	1.20 ± 0.88	1.51 ± 0.69	0.018
Fasting glucose (mg/dL)	114.43 ± 29.81	113.39 ± 29.38	118.54 ± 31.40	0.266
Albumin (g/dL)	4.14 ± 0.52	4.21 ± 0.53	3.88 ± 0.42	< 0.001
Triglyceride (mg/dL)	117.33 ± 65.46	122.39 ± 69.09	97.42 ± 43.69	0.002
LDL cholesterol (mg/dL)	92.61 ± 31.48	95.90 ± 32.12	76.79 ± 22.69	< 0.001
GGT (U/L)	133.45 ± 336.63	138.57 ± 371.10	112.42 ± 116.71	0.668
Prothrombin time (INR)	1.05 ± 0.12	1.03 ± 0.12	1.13 ± 0.12	< 0.001
hs-CRP (mg/dL)	0.42 ± 0.94	0.35 ± 0.73	0.65 ± 1.45	0.320
Child pugh class				0.330
Class A	240(93.39%)	193(94.15%)	47(90.38%)	
Class B	17(6.61%)	12(5.85%)	5(9.62%)	
Comorbidity				
Diabetes mellitus	92(35.80%)	71(34.63%)	21(40.38%)	0.440
Hypertension	88(34.24%)	78(38.05%)	10(19.23%)	0.011
Malignancy	17(6.61%)	9(4.39%)	8(15.38%)	0.004
Thyroid disease	9(3.50%)	8(3.90%)	1(1.92%)	0.488
Heart disease	11(4.28%)	10(4.88%)	1(1.92%)	0.347
Hematologic disease	5(1.95%)	3(1.46%)	2(3.85%)	0.267
Prophylaxis with endoscopic variceal ligation	61(23.74%)	30(14.63%)	31(59.62%)	< 0.001
Prophylaxis with beta-blocker	85(33.07%)	48(23.41%)	37(71.15%)	< 0.001
Compensated Advanced Chronic Liver Disease	112(43.58%)	89(43.41%)	23(44.23%)	0.916
Previously decompensated	53(20.62%)	30(14.63%)	23(44.23%)	< 0.001

VNT, varices needing treatment; HVPG, hepatic venous pressure gradient;

AST, aspartate aminotransferase; ALT, alanine aminotransferase; FBS, fasting blood sugar; LDL, low density lipid; GGT, gamma glutamyl transferase; INR, international normalized ratio; hs-CRP, high-sensitivity C-reactive protein;

Data are reported as means ± standard deviations for continuous variables and frequencies (%) for categorical variables.

### Factors related to the measurement failure of spleen stiffness

SSM failed in 15 out of 257 patients, resulting in a measurement success rate of 94.16%. Factors related to SSM failure were analyzed, and in multivariate analysis, the probability of SSM failure significantly increased when the spleen length was short (odds ratio [OR] 0.891, 95% CI 0.843–0.942, *P* < 0.001), the spleen volume was small (OR 0.961, 95% CI 0.943–0.980, *P* < 0.001), or the body mass index (BMI) was high (OR 1.143, 95% CI 1.003–1.330, *P* = 0.043) (Supplementary Table 1). Next, we derived cut-off values for each factor (Table 2). A spleen length less than 92.90 mm, a spleen volume less than 168.59 cm^3^, or a BMI higher than 24.17 kg/m^2^ were identified as cut-off points with a predicted probability of SSM failure of areas under the curve (AUC) 0.89, 0.94, and 0.65, respectively.

**Table 2:** Prediction of spleen stiffness measurement failure.

	Threshold	Se (%)	Sp (%)	Accuracy	PPV	NPV	AUC
Spleen length (mm)	92.90	1.00	0.74	0.76	0.18	1.00	0.89 (0.83–0.94)
Spleen volume (cm^3^)	168.59	1.00	0.83	0.84	0.26	1.00	0.94 (0.90–0.97)
Body mass index (kg/m^2^)	24.17	0.86	0.49	0.51	0.09	0.98	0.65 (0.53–0.74)

Se, sensitivity; Sp, specificity; PPV, positive predictive value; NPV, negative predictive value; AUC, area under the curve.

### Accuracy of pre-existing prediction models for VNT

First, we compared the ability to predict VNT of previously published non-invasive tests (NITs) (Table 3). The NITs used for comparison included platelet count, liver stiffness, spleen parameter (length or volume), LSPS, PSR, and VRS. Additionally, we evaluated the accuracy of the criteria for screening VNT in the recently published Baveno VII guideline. The sensitivity of the Baveno VII criteria defined as LSM and platelets was 0.96 with AUC 0.70 (0.66–0.75), missed VNT rate 2.15%, and spared endoscopy rate 44.39%. When combining Baveno VII with SSM applied to LSM and platelet standards, the spared endoscopy rate significantly increased to 82.92%, but sensitivity and missed VNT rate did not improve compared to the existing Baveno VII model.

**Table 3:** Accuracy of pre-existing prediction models for varices needing treatment.

	Threshold	Se	Sp	Accuracy	PPV	NPV	AUC	Missed VNT (%)	Spared endoscopy rate (%)
Platelet count (× 10^3^/mL)	141.5	0.86	0.57	0.63	0.34	0.94	0.76 (0.67–0.82)	4.76	56.58
Liver stiffness (kPa)	19.75	0.64	0.78	0.75	0.42	0.89	0.76 (0.68–0.81)	10.34	76.09
Spleen volume (mL)	376	0.74	0.81	0.80	0.50	0.92	0.82 (0.74–0.88)	7.22	81.46
Spleen length (mm)	112.3	0.80	0.70	0.72	0.40	0.93	0.80 (0.72–0.86)	6.54	69.26
LSPS	14.5	0.92	0.69	0.74	0.42	0.97	0.85 (0.81–0.90)	2.86	66.34
PSR	1157.4	0.88	0.66	0.70	0.39	0.96	0.79 (0.71–0.85)	4.32	33.65
VRS	52.2	0.78	0.76	0.76	0.44	0.93	0.83 (0.76–0.88)	6.63	72.68
Baveno VII criteria	LSM ≤ 20 kPa and platelet ≥ 150 k	0.96	0.44	0.54	0.30	0.98	0.70 (0.66–0.75)	2.15	44.39
Combined Baveno VII criteria	First, LSM < 20 kPa and PLT > 150, then if these criteria are not met, SSM < 40 kPa	0.85	0.83	0.83	0.56	0.96	0.84 (0.78–0.89)	4.49	82.92

Se, sensitivity; Sp, specificity; PPV, positive predictive value; NPV, negative predictive value; AUC, area under the curve; VNT, varices needing treatment; PSR, Platelet count/spleen diameter ratio; VRS, varices risk score; LSM, liver stiffness measurement; SSM, spleen stiffness measurement.

### Identifying a new cut-off of SSM for VNT

We identified a new cut-off of SSM that could improve the diagnostic performance while maintaining an accepted risk of missed VNT (< 5%). In the final analysis, SSM 38.9 kPa was presented as the new standard (Table 4). The 38.9 kPa cut-off improved sensitivity from 0.88 to 0.92 compared to the existing 40 kPa cut-off, reduced the missed VNT rate from 3.59 to 2.25%, and increased the spared endoscopy rate from 78.53 to 84.87% (McNemar test *p*-value 0.019). Additionally, the AUC was 0.88, which was higher than the existing 40 kPa AUC of 0.83 and was statistically significant (Delong test *p*-value 0.017). When analyzed separately into viral etiology and non-viral etiology, the SSM 38.9 kPa cut-off showed superior performance compared to the SSM 40 kPa cut-off in both viral etiology (Delong test *p*-value 0.019) and non-viral etiology (Delong test *p*-value 0.032). Next, a sensitivity analysis was performed targeting cACLD patients. There were a total of 112 patients with cACLD, and baseline characteristics are listed in Supplementary Table 2. In an analysis of 112 cACLD patients with an LSM of 10 kPa or higher, who had not experienced prior decompensation, the newly proposed SSM model (with a cut-off of 38.9 kPa) demonstrated superior efficacy compared to the Baveno VII criteria, which uses LSM and platelet count (DeLong test *p*-value < 0.001) (see Supplementary Table 3). While the difference in AUC between the cut-offs of 38.9 kPa and 40 kPa was not statistically significant, the cut-off of 38.9 kPa resulted in a slightly lower missed VNT rate (2.60% vs. 2.82%) and a higher rate of spared endoscopies (84.26% vs. 77.52%) than the 40 kPa cut-off.

**Table 4:** Cut-off of spleen stiffness for predicting varices needing treatment.

All (n = 257)	Se	Sp	Accuracy	PPV	NPV	AUC	P*	Missed VNT (%)	Spared endoscopy rate (%)	P**
Cut-off: SSM 40 kPa	0.88	0.78	0.81	0.51	0.96	0.83 (0.78–0.89)	0.017	3.59	78.53	0.019
Cut-off: SSM 38.9 kPa	0.92	0.85	0.86	0.60	0.98	0.88 (0.84–0.93)		2.25	84.87	
Viral (n = 119)	Se	Sp	Accuracy	PPV	NPV	AUC		Missed VNT (%)	Spared endoscopy rate (%)	
Cut-off: SSM 40 kPa	0.77	0.80	0.80	0.41	0.95	0.79 (0.68–0.90)	0.019	4.71	80.19	0.092
Cut-off: SSM 38.9 kPa	0.83	0.88	0.87	0.56	0.97	0.86 (0.76–0.95)		3.26	88.11	
Non-viral (n = 138)	Se	Sp	Accuracy	PPV	NPV	AUC		Missed VNT (%)	Spared endoscopy rate (%)	
Cut-off: SSM 40 kPa	0.94	0.77	0.81	0.57	0.98	0.86 (0.80–0.91)	0.032	2.44	78.84	0.219
Cut-off: SSM 38.9 kPa	0.97	0.82	0.86	0.63	0.99	0.90 (0.85–0.94)		1.23	82.69	

*P value for AUC.

**P value for spared endoscopy rate.

Se, sensitivity; Sp, specificity; PPV, positive predictive value; NPV, negative predictive value; AUC, area under the curve; VNT, varices needing treatment.

**Figure 2:**
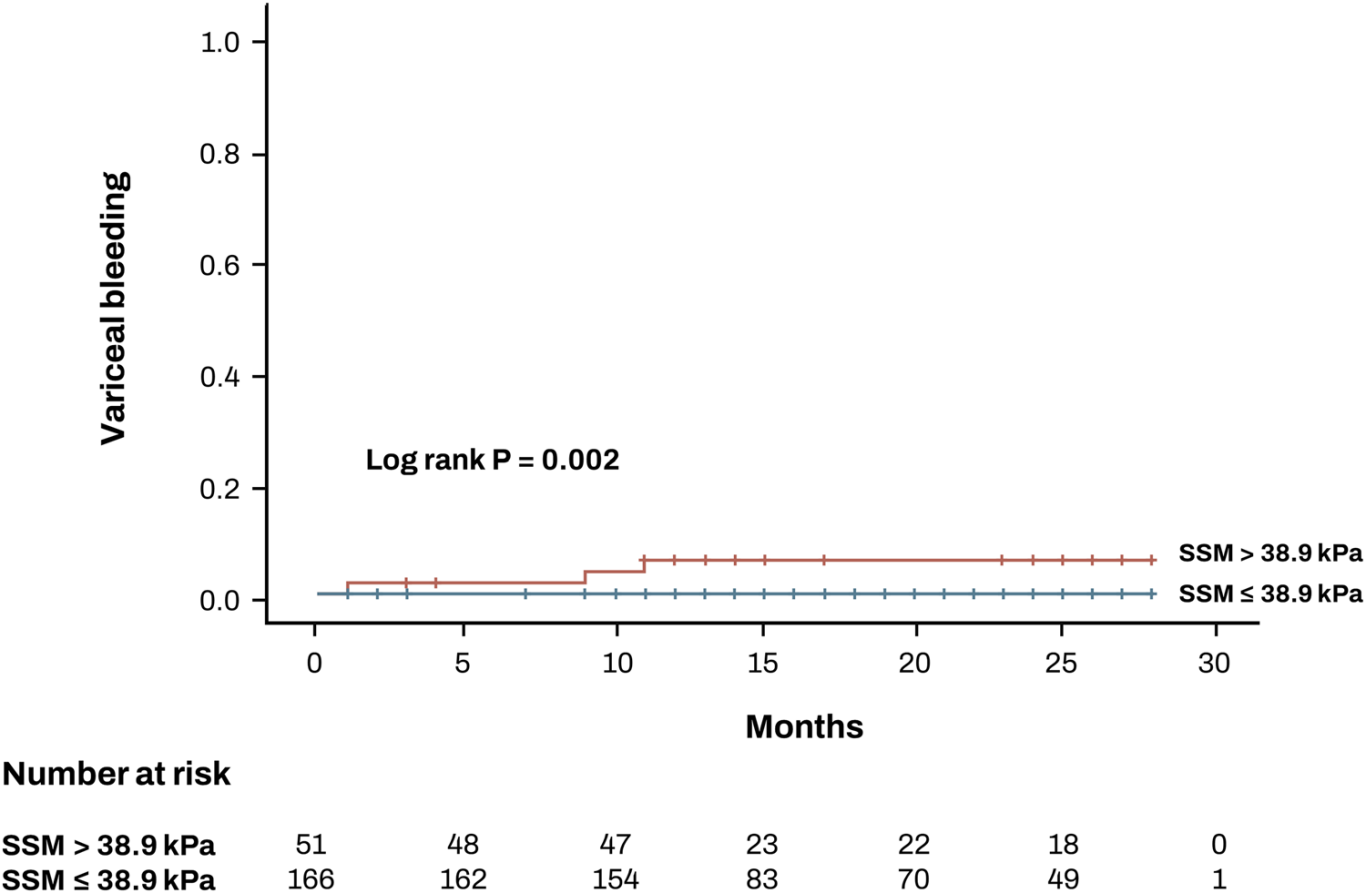
Incidence of new-onset variceal bleeding according to SSM cut-off.

The cut-off of 38.9 kPa for SSM was also effective in predicting future incidence of variceal bleeding. Upon follow-up over a median duration of 18 months, four patients with an SSM of 38.9 kPa or higher experienced variceal bleeding, whereas no patients with an SSM below 38.9 kPa did. Kaplan–Meier analysis further revealed a significant difference (Fig. 2, log rank *p* = 0.002).

### Spleen stiffness comparison with HVPG

**Figure 3:**
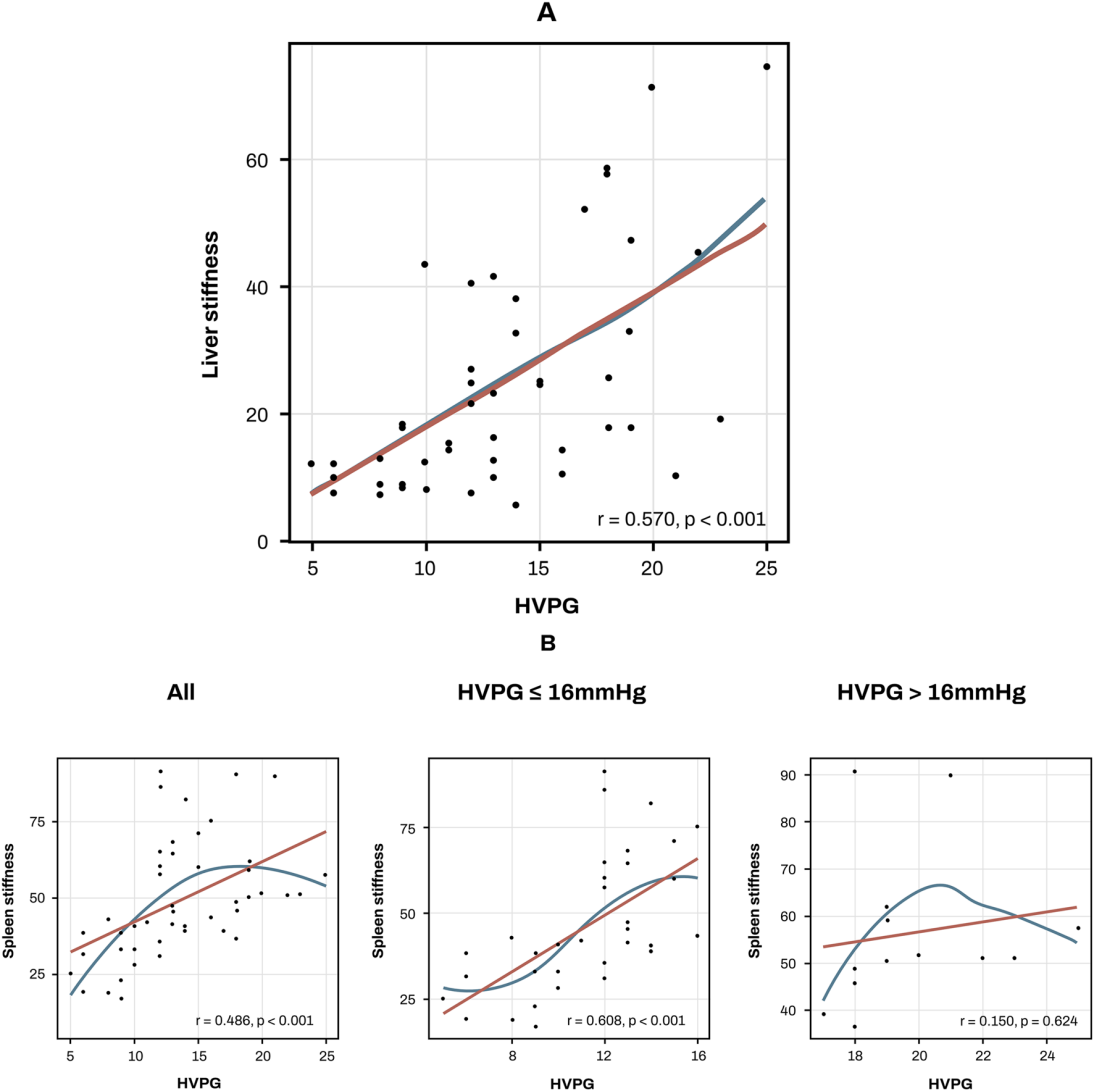
Pearson correlation analysis (**A**) between liver stiffness and HVPG, (**B**) between spleen stiffness and HVPG.

The correlation between HVPG and spleen stiffness or liver stiffness was analyzed for the 49 patients whose HVPG was measured. HVPG and liver stiffness (Fig. 3A, Pearson correlation coefficient 0.570, *P* < 0.001) or spleen stiffness (Fig. 3B, Pearson correlation coefficient 0.486, *P* < 0.001) showed a significant correlation. However, the scatterplot with a smooth line revealed that the correlation between HVPG and spleen stiffness was significant only at HVPG levels of 16 mm Hg or less (Fig. 3B). As a result, stratified analysis was conducted based on HVPG levels of 16 mm Hg. A significant correlation was observed between spleen stiffness and HVPG less than 16 mm Hg (Pearson correlation coefficient 0.608, *P* < 0.001), but this significance disappeared in HVPG levels of 16 mm Hg or higher (Pearson correlation coefficient 0.150, *P* = 0.624).

### Accuracy of pre-existing prediction models and new cut-off of SSM for CSPH

We analyzed how well the previously published NIT model and the new SSM cut-off of 38.9 kPA predicted CSPH (over 10 mm Hg of HVPG) (Table 5). The 38.9 kPa cut-off, which specifically derived and validated for diagnosing VNT, was also applied to predict CSPH. As expected, this cut-off value exhibited lower sensitivity and negative predictive value (NPV) for prediction of CSPH than when applied to VNT.” For SSM, cut-offs of 40 kPa, 50 kPa, and 38.9 kPa are used to rule in CSPH, showing sensitivities of 79%, 53%, and 84%, respectively, and specificities of 91%, 100%, and 90%. The accuracy for these cut-offs ranges from 63 to 86%, with the 38.9 kPa cut-off demonstrating the highest accuracy and area under the curve (AUC). The Baveno VII model outlines criteria for ruling out and ruling in CSPH with varying degrees of sensitivity, specificity, and AUC values.

**Table 5:** Accuracy of pre-existing prediction models for CSPH.

All (n = 49)	Threshold	Se	Sp	Accuracy	PPV	NPV	AUC
Spleen stiffness (cut-off:40 kPa)	To rule-in CSPH (SSM 40 kPa)	0.79	0.91	0.82	0.97	0.56	0.85 (0.74–0.96)
Spleen stiffness (cut-off:50 kPa)	To rule-in CSPH SSM 50 kPa	0.53	1.00	0.63	1.00	0.38	0.76 (0.68–0.84)
Spleen stiffness (cut-off:38.9 kPa)	To rule-in CSPH (SSM 38.9 kPa)	0.84	0.90	0.86	0.97	0.63	0.88 (0.77–0.98)
Baveno VII model	To rule-out CSPH (LSM of 15 kPa or less and platelet count of 150 × 10^9^ platelets per L or higher)	0.18	0.97	0.80	0.67	0.80	0.57 (0.46–0.70)
	To rule-in CSPH (LSM of 25 kPa or higher)	0.50	1.00	0.61	1.00	0.37	0.75 (0.67–0.83)
Baveno VII-SSM single cut-off model	To rule-out CSPH (at least two of the following criteria were present: LSM of 15 kPa or less, platelet count of 150 × 10^9^ platelets per L or higher, and SSM of 40 kPa or less)	0.72	0.86	0.83	0.62	0.92	0.80 (0.65–0.94)
	To rule-in CSPH (at least two of the following criteria were present: LSM of 25 kPa or higher, platelet count of less than 150 × 109 platelets per L, and SSM of greater than 40 kPa)	0.76	0.91	0.80	0.97	0.52	0.83 (0.72–0.94)
Baveno VII-SSM dual cut-off model	To rule-out CSPH (at least two of the following criteria were present: LSM of 15 kPa or less, platelet count of 150 × 10^9^ platelets per L or higher, and SSM of less than 21 kPa)	0.36	0.97	0.84	0.80	0.84	0.67 (0.52–0.82)
	To rule-in CSPH (at least two of the following criteria were present: LSM of 25 kPa or higher, platelet count of less than 150 × 10^9^ platelets per L, and SSM of greater than 50 kPa.)	0.58	1.00	0.67	1.00	0.41	0.79 (0.71–0.87)

Se, sensitivity; Sp, specificity; PPV, positive predictive value; NPV, negative predictive value; AUC, area under the curve; LSM, liver stiffness measurement; SSM, spleen stiffness measurement.

## Discussion

SSM began to gain attention relatively recently compared to LSM and is now an officially recommended tool in the 2021 Baveno VII guideline. However, the current evidence level remains relatively low at C2, with limited research findings, particularly regarding the optimal cut-off value of SSM for non-viral etiologies. The present study analyzed SSM@100 Hz and endoscopy data from 257 cirrhosis patients, providing insights into: (1) the success rates of SSM and factors associated with measurement failures, (2) the predictive ability of newly proposed SSM cut-off 38.9 kPa for VNT and CSPH compared to other NITs, and (3) the correlation between SSM and HVPG.

The first finding of our study is the success rate and factors related to SSM failure. The success rate using SSM@100 Hz in our study was 94.16%, significantly higher than the success rates reported for pSWE or 2D-SWE. SSM is known to be more challenging to measure than LSM, with some studies reporting success rates as low as 52.9%^8,9^. The success rate of SSM@100 Hz observed in the present study was comparable to the 95% success rate for LSM and 70% success rate for SSM reported in previous studies^10^. This success rate aligns well with recently published prospective studies^11,12^. The factors associated with SSM failure were consistent with those reported in prior research^13^, including small spleen length or volume and high BMI. Therefore, SSM might be less suitable for patients with normal spleen volume who have not progressed to advanced fibrosis. Obesity also affects SSM, as it does with LSM.

The second significant finding of our study was the efficacy of SSM@100 Hz as a predictive tool for VNT. Notably, the accuracy of SSM was higher than that of other NITs. There are two primary reasons for the higher accuracy of SSM. First, SSM is less affected by liver necroinflammation compared to LSM^14,15^. Second, SSM more directly reflects HVPG, making it a more accurate hemodynamic marker for acute changes compared to LSM^14,16,17^. In fact, SSM has shown to accurately predict hemodynamic response after NSBB administration, a prediction not achieved by LSM^18,19^. The clinical utility of SSM remained high even when different measurement methods or probes were used^5,20^. A meta-analysis of studies evaluating SSM with 2D-SWE and point-SWE found that SSM's predictive rate for high-risk esophageal varices (HREV) was AUC 0.87, and for any esophageal varices (E. varix) was AUC 0.90^4^, which is similar to the findings of our study. Both our study and the meta-analysis reported high negative predictive values (NPVs) for SSM, indicating its potential as a valuable screening tool to avoid unnecessary endoscopy^4,21,22^. In particular, our study verified the newly proposed cut-off SSM@100 Hz 38.9 kPa. Our study demonstrated a marginally enhanced performance of the SSM cut-off at 38.9 kPa compared to the conventional 40 kPa threshold. However, 1.1 kPa difference between two is small. Therefore, the existing Baveno criteria is still clinically relevant and useful.

Comparatively, our results resonate well with the burgeoning body of literature exploring the use of the 100-Hz SSM probe for liver cirrhosis patients. Notably, previous studies have similarly reported on the efficacy of SSM in predicting variceal outcomes, providing a consensus on the reliability of SSM measurements^20,23^. However, our study extends these findings by suggesting a refined cut-off point that potentially enhances diagnostic accuracy and patient care, a comparison that underscores the importance of continuous evaluation and adaptation of diagnostic thresholds. Moreover, our findings highlight the paramount importance of diagnosing CSPH over merely identifying VNT. This approach shifts the diagnostic focus towards a more holistic understanding of the patient's portal hypertension status, facilitating earlier and potentially more effective interventions.

While we emphasized the superior accuracy of SSM compared to NITs, we acknowledge the need to discuss other algorithms' performances as detailed in our methods and Table 3. Notably, the LSPS showed slightly higher accuracy than the combined Baveno VII-SSM model. However, despite similar accuracies of various models, the Baveno VII-SSM is supported by extensive evidence and validations, reinforcing its use in clinical guidelines.

The role of etiology in the diagnostic accuracy of SSM presents a nuanced view. Our study observed a low rate of patients with Hepatitis C Virus (HCV) infection, which contrasts with the demographic predominantly seen in older studies. This shift in patient demographics could reflect changes in disease prevalence or treatment outcomes over time. On the other hand, the lower rate of metabolic associated steatohepatitis liver disease (MASLD) patients in our cohort represents a limitation and points towards the need for further research encompassing a broader spectrum of liver diseases. Remarkably, the etiology did not influence the diagnostic accuracy of SSM in our study, a finding that emphasizes the robustness of SSM as a predictive tool across different liver disease etiologies.

Finally, our study demonstrated a correlation between SSM and HVPG. However, this correlation was more pronounced in the HVPG < 16 mm Hg group, and its significance decreased when HVPG was higher than 16 mm Hg. This observation is likely due to the development of various porto-systemic collaterals in cases of HVPG 16 mm Hg or higher, indicating severe portal hypertension or impending decompenstation. Stefanescu et al.^13^ also reported a high correlation between HVPG and SSM, but upon visual examination of the scatter plot, it becomes evident that the correlation diminishes at HVPG levels of 16 mm Hg or higher. Similar findings have been reported for the correlation between LSM and HVPG, showing a stronger association at lower HVPG levels^24,25^. However, given that HVPG was measured in only 49 patients in our study, it is essential to supplement these results with data from a larger number of patients in the future.

Our study has several strengths, including the use of a newly developed SSM@100 Hz probe and providing information on the cut-off and accuracy of SSM for non-viral etiology, which was previously lacking. Additionally, by presenting factors and criteria related to SSM measurement failure, which were not well-reported in other studies, we aimed to enhance the clinical utility of SSM. However, there are several limitations to consider. First, retrospective designs may introduce selection bias. Second, since only SSM@100 Hz was used in all patients, the criteria presented in this study may not be equally applicable to point-SWE or 2D-SWE.

In conclusion, our study not only reaffirms the efficacy of SSM@100 Hz in predicting the presence of VNT and variceal bleeding but also proposes a new diagnostic cut-off that could refine current practices. By drawing parallels with existing literature and addressing the significance of CSPH diagnosis and the impact of etiology, we contribute to the evolving landscape of non-invasive liver disease diagnostics.

## Materials and methods

### Patients

This retrospective cohort study was conducted from January 2020 to December 2022. The inclusion criteria for this study were as follows: (1) patients diagnosed with compensated liver cirrhosis by imaging or pathological examination, (2) age ranging from 19 to 70 years, and (3) esophagogastroduodenoscopy performed within 6 months from the time of SSM. Patients who had experienced previous episodes of decompensation but currently presented with compensated liver cirrhosis were included in the analysis. The exclusion criteria were as follows: (1) patients with decompensated cirrhosis at the moment of inclusion, (2) aspartate transaminase (AST) or alanine transaminase (ALT) levels exceeding 200 IU/L, (3) any grade of gastric varices, (4) previous history of transjugular intrahepatic portosystemic shunt, balloon-occluded retrograde transvenous obliteration, or plug-assisted retrograde transvenous obliteration, (5) patients with blood diseases that may affect SSM, (6) patients with difficulty in SSM due to severe obesity or ascites, and (7) patients with untreated or uncontrolled hepatocellular carcinoma and cholangiocarcinoma.^26,27^ The study protocol was approved by the Institutional Review Boards of Soonchunhyang University Bucheon Hospital (IRB number: SCHBC 2023-01-015, Date of registration: 03-Feb-2023). The study adhered to the ethical guidelines of the World Medical Association Declaration of Helsinki. Written consents were waived by the IRB of Soonchunhyang University Bucheon Hospital due to the retrospective nature of the study.

### Spleen stiffness measurement

SSM was performed using a newly developed 100 Hz probe (SSM@100 Hz) with the FibroScan® Expert 630 model (Echosens, France). After confirming the spleen hilum area with the ultrasound probe that came with the Fibroscan® machine, we placed the probe on the relevant area and measured the spleen stiffness 10 times consecutively. The SSM measurements were conducted by two physicians with extensive experience in over 100 SSM cases. The criteria used for spleen stiffness measurement were similar to those employed for liver stiffness evaluation, requiring a minimum of 10 measurements, a success rate of at least 60%, and an interquartile range (IQR) less than 30% of the median value^13,28,29^. Ultrasound examinations were performed to measure the longitudinal spleen length, thickness, and width by three experienced physicians with more than 10 years of pertinent experience. The spleen volume was calculated using the formula: π/6 × spleen length × thickness × width^30^.

### Endoscopy and HVPG

All patients underwent endoscopy. A standard esophagogastroduodenoscopy was performed by three experienced physicians with more than 10 years of experience. The endoscopic findings related to esophageal varices were documented, including the variceal grade and the presence of red signs.

If HVPG was measured within 6 months of SSM, HVPG values were also collected. The method for measuring HVPG was consistent with the protocol published previously^31,32^. Two experienced interventional radiologists, each with over a decade of expertise, conducted the HVPG measurements. The preferred access route was through the right jugular vein, where a 6-French balloon catheter was inserted into the right hepatic vein to measure the free hepatic venous pressure (FHVP). The wedge hepatic venous pressure (WHVP) was determined by inflating the balloon catheter within the right hepatic area. The HVPG was then derived by calculating the difference between the WHVP and the FHVP.

### Prediction models for VNT

VNT was defined as the presence of any of the following three conditions: (1) grade 1 esophageal varices with red color sign, or (2) medium to large esophageal varices (grade 2 or 3)^33,34^. Regarding VNT prediction, our study compared the following previously published scoring systems: LSM evaluated by the FibroScan® Expert 630 model, LSPS (platelet count to longitudinal spleen diameter ratio)^35^, PSR (platelet count/spleen diameter ratio)^36^, and VRS (varices risk score)^37^. Additionally, the recently published Baveno VII criteria using LSM and PLT criteria (LSM ≤ 20 kPa and platelet ≥ 150 k) or SSM single criteria (SSM ≤ 40 kPa) using SSM@100 Hz were also used for comparison^6^. The Combined Baveno VII criteria were defined as initially applying the criteria of LSM < 20 kPa and PLT > 150 k; if these criteria were not met, then applying SSM < 40 kPa was the next step. For VNT prediction, the analysis initially encompassed the entire patient group, followed by a sensitivity analysis conducted specifically on the compensated advanced chronic liver disease (cACLD) patient group. cACLD was defined as a patient having an LSM of 10 kPa or higher and no history of decompensation.

### Prediction models for CSPH

CSPH was defined as patients with HVPG of at least 10 mm Hg. Regarding CSPH prediction, we compared the following previously published scoring systems: LSM evaluated by the FibroScan® Expert 630 model, SSM single criteria (SSM ≤ 40 kPa), Baveno VII model, Baveno VII-SSM single cut-off model, and Baveno VII-SSM dual cut-off model^38^. In the Baveno VII model, CSPH was ruled out for patients with an LSM of 15 kPa or lower and a platelet count of at least 150 k, and ruled in for those with an LSM over 25 kPa. In the Baveno VII-SSM single cut-off model, CSPH was ruled out if at least two of the following criteria were met: an LSM of 15 kPa or lower, a platelet count of 150 k or higher, and an SSM of 40 kPa or lower; it was ruled in if at least two of these conditions were present: an LSM greater than 25 kPa, a platelet count less than 150 k, and an SSM over 40 kPa. The Baveno VII-SSM dual cut-off model applied the same criteria, with a cutoff of less than 21 kPa for SSM to rule out, and more than 50 kPa to rule in, CSPH.

### Statistical analysis

We assessed the effectiveness and performance of noninvasive tests using metrics including sensitivity, specificity, accuracy, positive and negative predictive values, AUC, missed VNT rate, and the rate of avoided endoscopies. The rate of missed VNT was defined as the number of patients having VNT divided by the number of patients who ruled out VNT.

Initially, we verified the efficacy of existing published parameters, such as the Baveno VII criteria, and subsequently introduced a new threshold aimed at enhancing diagnostic performance while keeping the risk of missed VNT below 5% (with a negative predictive value greater than 95%). The DeLong test was employed to compare the AUC across different prediction models. McNemar test was used to compare the rate of spared endoscopies by each criteria. Continuous baseline characteristics were presented as means (± standard deviations) and compared using Student’s t-test. Categorical characteristics were presented as counts and percentages and compared between groups using the chi-squared test. Logistic regression analysis was conducted to evaluate the factors related to SSM failure. Factors that showed significance in the univariate analysis were included in the multivariate analysis. Statistically significant differences were defined as *P* < 0.05. All statistical analyses were performed using R version 4.3.1 (The R Foundation for Statistical Computing, Vienna, Austria).

### Ethics declarations and Informed consent statement

The study protocol was approved by the Institutional Review Boards of Soonchunhyang University Bucheon Hospital (IRB number: SCHBC 2023-01-015, Date of registration: 03-Feb-2023). The study adhered to the ethical guidelines of the World Medical Association Declaration of Helsinki. Written consents were waived by the IRB due to the retrospective nature of the study.

### Supplementary Information

Supplementary Tables.

## Data Availability

The datasets generated during and/or analysed during the current study are available from the corresponding author on reasonable request.
