# Supplementary material for: Enhancing liver cirrhosis varices and CSPH risk prediction with spleen stiffness measurement using 100-Hz probe
Source: Sci Rep. 2024 Jun 13;14:13674. doi: 10.1038/s41598-024-63848-5 (PMC11176303; doi:10.1038/s41598-024-63848-5)
Supplement: Supplementary file 1 — Supplementary Tables. [file 41598_2024_63848_MOESM1_ESM.docx]

**Supplementary Table 1. Logistic regression analysis for spleen stiffness measurement failure**

|  | **Univariable** |  | **Multivariable 1** |  | **Multivariable 2** |  |
| --- | --- | --- | --- | --- | --- | --- |
|  | **OR (95% CI)** | **p** | **OR (95% CI)** | **p** | **OR (95% CI)** | **p** |
| **Male** | 0.585 (0.206-1.667) | 0.316 |  |  |  |  |
| **Age (years)** | 1.048 (0.993-1.105) | 0.087 |  |  |  |  |
| **spleen length (mm)** | 0.891 (0.843-0.942) | <0.001 | **0.839 (0.772-0.912)** | **<0.001** |  |  |
| **spleen volume (mL)** | 0.961 (0.943-0.980) | <0.001 |  |  | **0.950 (0.927-0.974)** | **<0.001** |
| **Etiology: viral (vs. non-viral)** | 2.440 (0.810-7.353) | 0.113 |  |  |  |  |
| **Body mass index** | 1.143 (1.003-1.330) | 0.043 | **1.535 (1.167-2.018)** | **0.002** | **1.585 (1.158-2.171)** | **0.004** |
| **Diabetes** | 0.891 (0.295-2.690) | 0.838 |  |  |  |  |
| **Liver stiffness (kPa)** | 0.983 (0.944-1.025) | 0.430 |  |  |  |  |
| **Esophageal varices any grade** | 0.931 (0.321-2.697) | 0.895 |  |  |  |  |
| **Varices need treatment** | 0.268 (0.034-2.082) | 0.208 |  |  |  |  |
| **Hepatic vein pressure gradient** | 0.665 (0.316-1.397) | 0.281 |  |  |  |  |
| **Platelet** | 1.007 (1.001-1.012) | 0.020 | 1.000 (0.993-1.008) | 0.940 | 1.000 (0.992-1.009) | 0.944 |
| **Child-pugh class** |  |  |  |  |  |  |
| Child-pugh class A | 1 (ref) |  |  |  |  |  |
| Child-pugh class B | 0.312 (0.037-2.532) | 0.998 |  |  |  |  |

**Supplementary Table 2. Baseline characteristics of compensated advanced chronic liver disease (cACLD)**

|  | **Total**  **(N = 112)** |
| --- | --- |
|  |  |
| **Age (years)** | 59.81 ± 10.51 |
| **Sex, male** | 61(54.46%) |
| **Etiology** |  |
| HBV | 46(41.07%) |
| HCV | 4(3.57%) |
| Alcohol | 38(33.93%) |
| NAFLD | 5(4.46%) |
| Others | 19(16.96%) |
| **Body mass index (kg/m²)** | 25.06 ± 3.87 |
| **Spleen stiffness measurement fail** | 7(6.25%) |
| **Spleen length (mm)** | 108.60 ± 20.21 |
| **Spleen volume (cm^3^)** | 322.13 ± 196.63 |
| **Spleen stiffness (kPa)** | 35.39 ± 17.09 |
| **Liver stiffness (kPa)** | 23.80 ± 16.39 |
| **HVPG (mmHg) (N=20)** | 13.75 ± 5.02 |
| **Platelet count (×10³/mL)** | 141.27 ± 75.58 |
| **AST (U/L)** | 49.35 ± 49.63 |
| **ALT (U/L)** | 32.75 ± 43.86 |
| **Total bilirubin (mg/dL)** | 1.37 ± 1.07 |
| **Fasting glucose (mg/dL)** | 115.43 ± 33.10 |
| **Albumin (g/dL)** | 4.08 ± 0.52 |
| **Triglyceride (mg/dL)** | 116.94 ± 61.22 |
| **LDL cholesterol (mg/dL)** | 90.92 ± 34.41 |
| **GGT (U/L)** | 177.93 ± 437.96 |
| **Prothrombin time (INR)** | 1.05 ± 0.11 |
| **hs-CRP (mg/dL)** | 0.33 ± 0.73 |
| **Child pugh class** |  |
| Class A | 105(93.75%) |
| Class B | 7(6.25%) |
| **Prophylaxis with endoscopic variceal ligation** | 17(15.18%) |
| **Prophylaxis with beta-blocker** | 31(27.68%) |

* Abbreviation: VNT, varices needing treatment; HVPG, hepatic venous pressure gradient;

AST, aspartate aminotransferase; ALT, alanine aminotransferase; FBS, fasting blood sugar; LDL, low density lipid; GGT, gamma glutamyl transferase; INR, international normalized ratio; hs-CRP, high-sensitivity C-reactive protein;

* Data are reported as means ± standard deviations for continuous variables and frequencies (%) for categorical variables.

**Supplementary Table 3.** **Accuracy of prediction for VNT in patients with cACLD**

|  | **Threshold** | **Se** | **Sp** | **Accuracy** | **PPV** | **NPV** | **AUC** | **Missed VNT (%)** | **Spared endoscopy rate (%)** |
| --- | --- | --- | --- | --- | --- | --- | --- | --- | --- |
| **Baveno VII criteria (LSM and Platelet)** | LSM≤20 kPa and platelet≥150 k | 0.96 | 0.30 | 0.44 | 0.26 | 0.96 | 0.63 (0.57-0.69) | 3.57 | 30.33 |
| **Baveno VII criteria (SSM only)** | SSM≤40 kPa | 0.91 | 0.78 | 0.80 | 0.51 | 0.97 | 0.84 (0.77-0.92) | 2.82 | 77.52 |
| **Combined Baveno VII criteria** | First, LSM <20 kPa and PLT >150, then if these criteria are not met, SSM <40 kPa | 0.87 | 0.79 | 0.80 | 0.51 | 0.96 | 0.83 (0.75-0.91) | 4.11 | 78.65 |
| **Newly proposed SSM model** | SSM≤38.9 kPa | 0.91 | 0.84 | 0.86 | 0.60 | 0.97 | 0.88 (0.81-0.95) | 2.60 | 84.26 |

*Abbreviation: Se, sensitivity; Sp, specificity; PPV, positive predictive value; NPV, negative predictive value; AUC, area under the curve; ; VNT, varices needing treatment; PSR, Platelet count/spleen diameter ratio; VRS, varices risk score; LSM, liver stiffness measurement; SSM, spleen stiffness measurement
